# Synthesis of Alkyl-Glycerolipids Standards for Gas Chromatography Analysis: Application for Chimera and Shark Liver Oils

**DOI:** 10.3390/md16040101

**Published:** 2018-03-23

**Authors:** Michelle Pinault, Cyrille Guimaraes, Hélène Couthon, Jérôme Thibonnet, Delphine Fontaine, Aurélie Chantôme, Stephan Chevalier, Pierre Besson, Paul-Alain Jaffrès, Christophe Vandier

**Affiliations:** 1 Inserm, UMR1069, Université de Tours, 37000 Tours, France; Michelle.pinault@univ-tours.fr (M.P.); cyrille.guimaraes@gmail.com (C.G.); delphine.fontaine@etu.univ-tours.fr (D.F.); aurelie.chantome@univ-tours.fr (A.C.); stephan.chevalier@univ-tours.fr (S.C.); pierre.besson@univ-tours.fr (P.B.); 2 CEMCA, CNRS UMR6521, Université de Brest, IBSAM, 6 Av V. Le Gorgeu, 29238 Brest, France; helene.Couthon@univ-brest.fr (H.C.); Paul-Alain.Jaffres@univ-brest.fr (P.-A.J.); 3 Equipe SIMBA, Synthèse et Isolement de Molécules BioActives, EA 7502, Université de Tours, 37000 Tours, France; jerome.thibonnet@univ-tours.fr; 4 Faculté de Sciences et Techniques, Université de Tours, 37000 Tours, France; 5 Faculté de Pharmacie, Université de Tours, 37000 Tours, France

**Keywords:** ether-lipids, alkyl-glycerolipids, standards for gas chromatography, fish oil

## Abstract

Natural *O*-alkyl-glycerolipids, also known as alkyl-ether-lipids (AEL), feature a long fatty alkyl chain linked to the glycerol unit by an ether bond. AEL are ubiquitously found in different tissues but, are abundant in shark liver oil, breast milk, red blood cells, blood plasma, and bone marrow. Only a few AEL are commercially available, while many others with saturated or mono-unsaturated alkyl chains of variable length are not available. These compounds are, however, necessary as standards for analytical methods. Here, we investigated different reported procedures and we adapted some of them to prepare a series of 1-*O*-alkyl-glycerols featuring mainly saturated alkyl chains of various lengths (14:0, 16:0, 17:0, 19:0, 20:0, 22:0) and two monounsaturated chains (16:1, 18:1). All of these standards were fully characterized by NMR and GC-MS. Finally, we used these standards to identify the AEL subtypes in shark and chimera liver oils. The distribution of the identified AEL were: 14:0 (20–24%), 16:0 (42–54%) and 18:1 (6–16%) and, to a lesser extent, (0.2–2%) for each of the following: 16:1, 17:0, 18:0, and 20:0. These standards open the possibilities to identify AEL subtypes in tumours and compare their composition to those of non-tumour tissues.

## 1. Introduction

**Figure 1:**
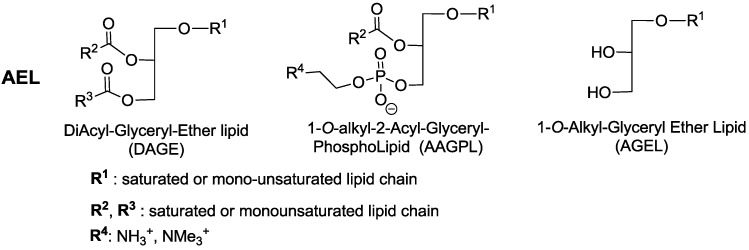
Chemical structure of AEL (alkyl-ether-lipid) subtypes: DAGE, AAGPL and the structure of the common fragment 1-*O*-alkyl-glyceryl ether lipid (AGEL). For simplification purposes, chirality is not depicted.

Natural *O*-alkyl-glycerolipids, also known as alkyl-ether-lipids (AEL), have a long fatty alkyl chain linked to the glycerol unit by an ether bond [1]. The most prevalent glycerol-backboned AEL found in nature consist of an *O*-alkyl or *O*-alk-1′-enyl group attached to glycerol at position *sn*-1. In cells, AEL can be triglycerides (diacyl-glyceryl-ether lipids, DAGE) which possess two fatty acyl moieties at positions *sn*-2 and *sn*-3, or phospholipids (1-*O*-Alkyl-2-Acyl-*sn*-glycerylphospholipids, AAGPL) with one fatty acyl chain at the *sn*-2 position and one phosphodiester group (e.g., phosphocholine, phosphoethanolamine). The common feature of these two series of compounds is the 1-*O*-alkyl-glyceryl ether lipid (AGEL), as shown in Figure 1.

Natural AEL were found to stimulate the immune system [2] and hematopoiesis [3]. They also have anti-tumor properties [4], reduce the side effects induced by radiotherapy [5], and lead to a reduction in the growth of tumor cells and metastases [6]. Interestingly, ether-lipids have been also considered for potential use in the diagnosis of prostate cancer [7].

Some biological effects of these AEL can be explained by an interaction with their receptors, such as the platelet-activating factor (1-*O*-alkyl-2-acetyl-*sn*-glycero-3-phosphocholine) [8,9], or following their integration in the phospholipids of cell membranes [10], and by a modification of the activity of Protein Kinase C (PKC) [11,12,13] which has a high activity in breast tumors [14]. The integration of AEL into the membrane results in the formation of DAG ether-lipid analogs, alkyl-acyl-glycerols, which inhibit PKC activation unlike the “classical” ester-linked, PKC-activating diacyl-glycerol DAG [12,15]. In addition to a decrease in PKC activity, it has been shown that these lipids inhibit the activity of other kinases such as phosphatidylinositol-3-kinase [16] and MAP kinases [17], but also Na^+^/K^+^ ATPase [18]. AEL derivative edelfosine (1-*O*-octadecyl-2-*O*-methyl-*sn*-glycero-3-phosphocholine) was found to inhibit the activities of Na^+^/H^+^ exchanger [19] and K^+^ channel [20], as well as the transport of nutrients [21]. Thus, all of these effects can participate in the anti-proliferative effects of AEL. The replacement of the phosphocholine moiety by an aminoglycoside group led to a large series of compounds exhibiting anticancer properties [22,23]. Interestingly, with the replacement of the phosphocholine moiety of edelfosine by a saccharide unit to design glyco-AEL, we identified less toxic compounds whereas the modulation of ion channels was preserved [24,25,26,27]. Among disaccharide-AEL, [28] we identified ohmline (1-*O*-hexadecyl-2-*O*-methyl-*rac*-glycero-3-lactose) as a lead synthetic glycol-AEL that can prevent metastasis development by modifying the activity of ion channel complexes (KCa-Ca^2+^) located in cholesterol-rich nano-domain and regulating calcium signaling and migration of cancer cells [29,30,31]. Some aspects of the mechanism of action of ohmline were recently reported, showing that ohmline fluidified the plasma membrane, especially if this plasma membrane had a high level of cholesterol [32]. Altogether, these results pointed out the great therapeutic potential of synthetic ether lipids and contribute to questions about the role of natural ether lipids. AEL are abundant in many tissues, and especially in shark liver oil, breast milk, red blood cells, blood plasma, and bone marrow [33,34,35]. Among human tissues, bone marrow contained the highest percentage of alkyl-glycerols in both triglycerides and phospholipids fractions [35]. In mammals, there is a higher prevalence of alkyl-glycerols in phospholipid fractions compared to neutral lipid (triglycerides) fractions [36]. Interestingly, tumors have been found to contain larger amounts of AEL than normal tissues [36], but the physiological roles of these natural AEL have not been elucidated so far. The clinical significance of their accumulation in tumors remains to be determined. Furthermore, no quantitative analysis has been carried out in tissues to precisely determine the proportion of AEL molecular species based on their alkyl chain length and unsaturation level. If some AELs are commercially available (e.g., 1-*O*-hexadecyl-*sn*-glycerol, also known as chimyl alcohol, and 1-*O*-octadecyl-*rac*-glycerol also known as batyl alcohol), others with saturated alkyl chain of variable length or monounsaturated alkyl chains are, unfortunately, not commercially available. These compounds are necessary as standards for analytical methods. This led us to investigate reported synthesis procedures and to adapt some of them to prepare a series of 1-*O*-alkyl-glycerols with saturated chain length (14:0, 16:0, 17:0, 19:0, 20:0, 22:0) and two monounsaturated chains (16:1, 18:1). All of these standards were fully characterized by NMR and GC-MS. Finally, we used these standards to precisely characterize alkyl-glycerols subtypes in shark and chimera liver oils.

## 2. Results and Discussion 

### 2.1. Alkyl-Glycerolipids Chemical Synthesis

**Figure 2:**
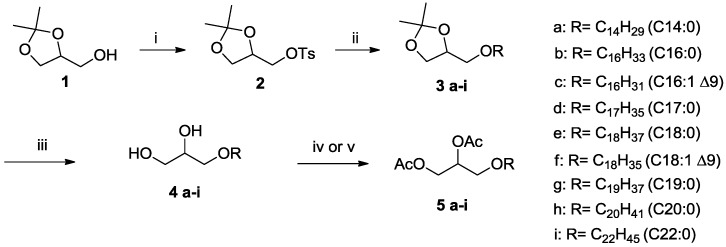
Synthesis of alkyl-glycerolipids **4a**–**i** and derivatization as diacetyl esters **5a**–**i**. (**i**) TsCl, Pyridine, 1 h, 0 °C then 24 h, rt; (**ii**) preparation of the alcoholate: NaH, ROH, dimethoxyethane, 1 h, then addition of **2**, 100 °C then 48 h, reflux; (**iii**) HCl 2N, Ethanol, 4 h, reflux. Method A (**iv**): acetic anhydride, pyridine, 60 °C, 30 min. and then 1 night at 20 °C; Method B (**v**): acetyl chloride, 5 min. 140 °C.

Since our goal was to have alkyl-glycerol standards for analytical purpose, the chirality at the sn-2 position was not an issue since the targeted compounds (**4a**–**i**; Figure 2) possess only one asymmetric carbon and, therefore, on non-chiral support, both enantiomers will possess similar physico-chemical properties including retention time. As a consequence, the syntheses of the standards were achieved in racemic series. Solketal **1** is the common commercial precursor to prepare *O*-alkyl glycerol **4** since the free alcohol function can be deprotonated and then engaged in an alkylation reaction with a lipid alcohol activated with a mesyl functional group or directly with a halogenoalkyl. Prinz et al. [37] used KOH to deprotonate solketal in benzene and then myristoylmethanesulfonate (C14:0) or palmitoylmethanesulfonate (C16:0) was added. After alcohol deprotection with HCl in methanol, the alkylglycerol **4a** (C14:0) and **4b** (C16:0) were isolated in 77–85% yield (two steps). Aranda et al. [38] only changed the solvent (DMSO) to prepare compound **4a** (C14:0) in 80–85% yield. Huang et al. [39] used NaH in toluene for deprotonation of solketal and a subsequent alkylation was achieved with bromoalkane (1-Bromotetradecane, 1-Bromohexadecane or 1-Bromooctadecane), thus producing compounds **4a**–**b** (yields were not reported). Another approach that retained our attention consisted of using lipid alcohol and racemic solketal **2** activated with a tosyl group as the leaving group. The use of this activated solketal was previously reported by Bhatia et al. [40] to produce 1-*O*-hexadecyl-*sn*-glycerol in 65% yield (including the acetal deprotection). We prepared the tosylate **2** from solketal **1** following a reported procedure [41]; it is worth noting that compound **2** is also commercially available (analytical data are available in the Supporting Material, SI1.7, 1.8). Then, we used the method of Bhatia et al. to introduce the alkylether moiety (Figure 2). First, the alkyl alcoholate was prepared by deprotonation of the alcohol with NaH and then this alcoholate reacted with the tosylated solketal **2** [42]. After work-up, the expected compounds **3a**–**i** were isolated in 60–96% yield. Then the deprotection of the acetal function was achieved in acidic conditions (HCl 2N) in methanol at reflux and the final compounds **4a**–**i** were isolated in 92–98% yield. If most of these compounds were reported and prepared by other methods, it must be noted that to the best of our knowledge, compounds **3/4c**, **3/4d**, **3/4g** were not reported before.

For the analysis of the 1-*O*-alkyl glycerol by GC, protection of the alcohol function must be achieved. For this derivation step, we have protected the alcohol function with acetate groups. For this purpose, two methods were employed. Method A consisted to use acetic anhydride in pyridine at 60 °C [43] and method B used acetyl chloride without solvent at 140 °C (Figure 2) [44]. Method A used mild conditions that should reduce degradation but the work-up (treatment with acid and basic solutions) induced sample losses. Method B is much faster and was efficient with a simplified work-up. However, this method requires heating at 140 °C that can induce, depending on the structure of the lipid chain, some degradation. In this series of compounds, no degradation was observed including for the mono-unsaturated compound **5c** and **5f**. The final compounds **5a**–**i** were fully characterized and isolated in good yield (92–98%). 

### 2.2. Liver Oils Alkyl-Glycerolipids Analysis

In order to precisely identify the alkyl-glycerol subtypes in shark and chimera liver oils we first extracted the total lipids of chimera and shark liver oil and derived alkyl-glycerols to alkyl-glycerol diacetates to be analyzed using gas chromatography as described in Section 3.2 and Section 3.4. 

**Figure 3:**
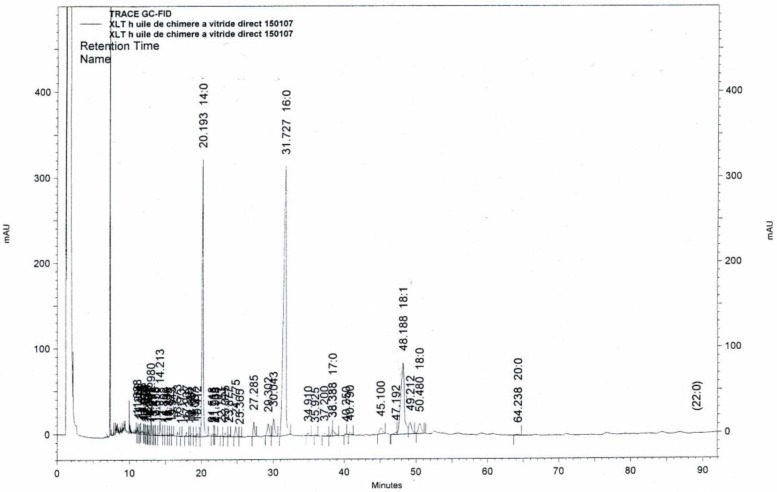
Chromatogram of alkyl-glycerols diacetate from chimera (*Chimaera monstrosa*) liver oil.

Figure 3 shows an example of chromatogram of alkyl-glycerols diacetate obtained from chimera (*Chimaera monstrosa*) liver oil. 

**Figure 4:**
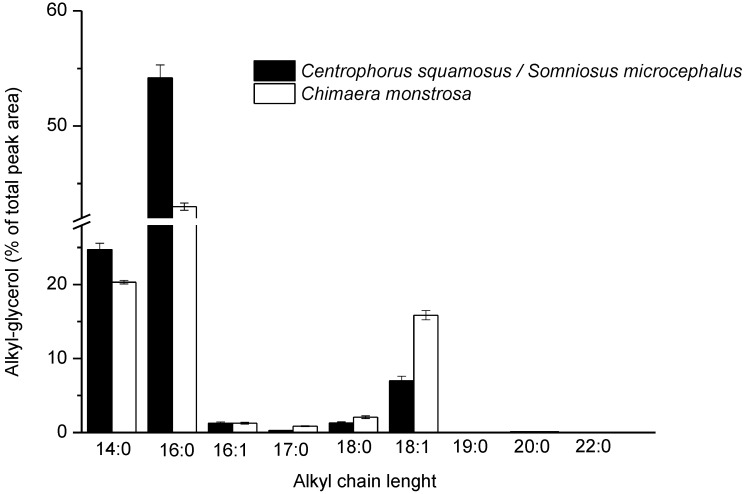
Comparative analysis of alkyl-glycerols of chimera (*Chimaera monstrosa*) and shark liver (a mixture of two species: *Centrophorus squamosus* and *Somniosus microcephalus*). Bars represent mean value and error bars represent SD, *n* = 6. Neither C19:0 nor C22:0 alkyl chains were detected.

Using the alkyl-glycerols standards, we analysed the alkyl-glycerol subtypes in shark and chimera liver oils (Figure 4). We found that both oils contain mainly alkyl-glycerols with C14:0, C16:0, and C18:1 alkyl chains and low amounts of C16:1 and C18:0 alkyl chains. Interestingly, C17:0 and C20:0 were found in *Chimaera monstrosa* and shark liver oils. Note, that we did not find C19:0 or C22:0 alkyl chains.

After derivatization as acetate, we optimized the temperature program for the separation and identification of alkyl-glycerolipids by GC-MS (see Supplementary Materials SI2.1, SI2.2). This method confirmed the results obtained using GC-FID.

We found the presence of 14:0 (20–24%), 16:0 (42–54%), and 18:1 (6–16%) alkyl-glycerols and, to a lesser extent (0.2–2%), 16:1, 17:0, 18:0 and 20:0 alkyl-glycerols. It was generally reported that liver oil of certain shark species contain high levels of alkyl-glycerolipids as a mixture of subtype that varied by length and unsaturation of the alkyl chain with almost exclusively saturated and monounsaturated, the main alkyl moieties being the C16 and C18 chains in both lipid classes [45,46]. Interestingly, a high level of 18:1 was also found in the composition of alkyl chains in alkyl-glycerolipids from Greenland shark (*Centrophorus squamosus*) liver oil [47]. Thus, our standards confirm the presence of these alkyl-glycerolipids in shark and chimera liver oils.

## 3. Materials and Methods

### 3.1. Chemical Synthesis

Chemicals and solvents ACS grade or higher, commercially available, were used without further purification (Carlo Erba, Val de Reuil, France; and Sigma-Aldrich, Saint-Quentin Fallavier, France). NMR spectra (Bruker, Wissembourg, France) were recorded on a Bruker DPX Avance 300 spectrometer (300 MHz for ^1^H, 75 MHz for ^13^C), Bruker DRX-500 (500 MHz for ^1^H, 125 MHz for ^13^C), and Bruker Ascend-600 AVANCE III HD equipped with TCI cryoprobe (600 MHz for ^1^H, 150 MHz for ^13^C). CDCl_3_ was used as the solvent; chemical shifts are expressed in ppm relative to TMS as an internal standard. Purification of crude products was carried out using Puriflash^®^ 430 (Interchim, Montluçon, France) equipped with an evaporative light-scattering detector (ELSD). Preparative thin-layer chromatography (TLC, LK5) separation was carried out using 20 × 20 cm, glass-backed, 1 mm or higher thickness silica gel Merck F_254_ plates (VWR, Fontenay-sous-Bois, France). Control TLC was performed using Merck F_254_ aluminium-backed silica gel sheets (VWR). Lipid compounds on TLC plates/sheets were revealed by 2′,7′-dichlorofluorescein. 

HMRS analysis was performed on the HRMS CNRS ICOA platform at the University of Orleans.

### 3.2. Analytical Chemistry

Chemicals and solvents, ACS grade or higher, commercially available, were used without further purification (Carlo Erba). 

After the extraction of total lipids by the Folch method [48] ester bonds were reduced using the strong reducing agent Vitride^®^ (sodium hydride and aluminum) (0.5 mL Red-Al^®^ for 10 mg total lipids) according to a described method [49], in a 80/20 *v/v* mixture of diethyl ether/hexane, giving ether-lipids (1-*O*-alkylglycerols and 1-*O*-alk-1-enyl-glycerols) and fatty alcohols. The reaction medium was neutralized at melting-ice temperature using ethanol/water (20/80, *v/v*). The reaction products were recovered from the diethyl ether phase and evaporated to dryness, then were diluted in chloroform/methanol (2/1, *v/v* ), and finally separated by TLC on 20 × 20 silica gel plate (LK5) with chloroform/acetone (90/10, *v/v* ) as the eluent. The plate was allowed to dry and was then sprayed with 2,7′dichlorofluorescein (0.2% in ethanol) to reveal the compounds under UV light. In parallel, batyl alcohol (C18:0 AGEL) was deposited on the same plate as a reference standard for collecting ether lipid samples at the same Rf = 0.18. The silica gel containing the ether lipids was scraped and transferred into a tube to be derivatized as 1-*O*-alkylglycerol-diacetate. Acetylation was performed with a large excess (600 µL) of ethanoyl chloride directly on the silica contained in the tube, by heating at 140 °C for 5 min. Only 1-*O*-alkylglycerol-diacetate were obtained since alkenyl lipids are destroyed by the presence of acid in the reaction. Reaction medium was neutralized with a saturated aqueous solution of sodium hydrogen carbonate. Diacetate of 1-*O*-alkylglycerols were extracted with diethyl ether, evaporated to dryness and dissolved in hexane before analysis by gas chomatography. Gas chromatographic separation of acetate-derivatized 1-*O*-alkylglycerolipids was performed on a Shimadzu GC-2010 Plus system (automatic liquid sample injection unit, on-column injector, flame ionization detector set at 325 °C) equipped with a Zebron 5-MS column (Phenomenex, Le Pecq, France), (30 m, 0.25 mm ID, 0.25 µm film thickness). The temperature program was set as follows: 60 °C (2 min. hold), to 180 °C at 14 °C/min (4 min. hold), to 185 °C at 0.1 °C/min (4 min. hold), then to 250 °C at 2 °C/min (25 min. hold). Hydrogen was used as a gas vector at constant pressure of 180 kPa.

### 3.3. Mass Spectrometry

#### Optimization of the Separation Method

GC-Ultra-ISQ (Thermo Fisher, Villebon sur Yvette, France) was used with a Zebron 5-MS column (Phenomenex, Le Pecq, France) having a 30 m length, 0.25 mm of diameter, and 0.25 μm film thickness. The injector temperature was 250 °C, the splitless injection volume was 1 μL, and the vector gas was hydrogen with a constant flow rate of 1.5 mL∙min^−1^. The temperature program was as follows: 140 °C for 1 min, followed by a ramp at 7·°C∙min^−1^ up to 310 °C, then a 3 min hold. The transfer line temperature was 310 °C. The source temperature was 300 °C. The solvent delay was 3 min and the mass range 35–500 *m*/*z*.

### 3.4. Experimentation Section

#### 3.4.1. Preparation of the (2,2-Dimethyl-1,3-dioxolan-4-yl)methyl 4-methylbenzenesulfonate (**2**)

To a cold (0 °C) solution of (±)-2,2-dimethyl-4-hydroxymethyl-1,3-dioxolane(4.0 g, 30 mmol), in anhydrous pyridine (6 mL), *p*-TsCl (7.0 g, 37 mmol) was added. After stirring for 24 h, diethyl ether (200 mL) was added and the solution was washed with aqueous HCl (1 M, 2 × 200 mL), H_2_O (5 × 100 mL), dried over Na_2_SO_4_, and filtered. Diethyl ether was evaporated to give the desired product as a clear oil. Yield = 81%. ^1^H NMR-CDCl_3_, δ (ppm) 7.80 (d, *J* = 8.3 Hz, 2H, *CH_Ar_*), 7.36 (d, *J* = 8.0 Hz, 2H, *CH_Ar_*), 4.34–4.22 (m, 1H, *CH*), 4.10–3.92 (m, 3H, *CH*), 3.77 (dd, *J* = 8.8, 5.1 Hz, 1H, *CH*), 2.46 (s, 3H, *ArCH_3_*), 1.34 (s, 3H, *CH_3_ acetate*), 1.31 (s, 3H, *CH_3_ acetate*).

#### 3.4.2. Preparation of 4-Alkoxymethyl-2,2-dimethyl-1,3-dioxolanes (**3a**–**i**)

Alcohol (1.0 eq.) and sodium hydride (60% in mineral oil, 2.8 eq.) were introduced in dimethoxyethane (10 mL). The solution was refluxed up to the end of H_2_ emission. Then, a solution of (2,2-dimethyl-1,3-dioxolan-4-yl)methyl 4-methylbenzenesulfonate (1.4 eq.) in ethane-1,2-diol was added and the mixture was refluxed for 48 h. After cooling to room temperature, the solution was poured into water. The organic layer was extracted with diethyl ether and dried over Na_2_SO_4_. The solvents were removed under vacuum and the desired product was obtained after a purification by flash chromatography in chloroform. The advancement of the purification was followed by TLC in chloroform/acetone (90:10).

**4-(*n*-Tetradecyloxymethyl)-2,2-dimethyl-1,3-dioxolane (3a)**. This product was obtained from 1-tetradecanol (0.5 g; 2.4 mmol). TLC performed in chloroform/acetone (90:10). Rf = 0.68. Yield = 68% (0.52 g). ^1^H NMR-CDCl3, δ (ppm) 4.26 (1H, m, *CH-glycerol*), 4.01 (1H, m, *CH_2_O*-*alkyl*), 3.76 (1H, m, *CH_2_O-alkyl glycerol*), 3.40–3.30 (4H, m, *CH_2_OC* and *CH_2_O-glycerol*), 1.70–1.50 (2H, m, *CH_2_CH_2_O-glycerol*), 1.48 (3H, s, C*H*_3_ *acetal*), 1.41 (3H, s, C*H*_3_ *acetal*), 1.29 (22H, m, *CH_2_*), 0.91 (3H, t, *J* = 6.8 Hz, *CH_3_*). ^13^C NMR-CDCl3_,_ δ (ppm) 109.6, 74.2, 71.8, 71.3, 67.3, 31.7, 29.7, 29.2, 29.0, 26.6, 25.7, 23.2, 14.0. 

**4-(*n*-Hexadecyloxymethyl)-2,2-dimethyl-1,3-dioxolane (3b).** This product was obtained from 1-hexadecanol (0.5 g; 2.1 mmol). TLC performed in chloroform/acetone (90:10). Rf = 0.68. Yield = 96% (0.71 g). ^1^H NMR-CDCl3, δ (ppm) 4.26 (1H, m, *CH-glycerol*), 4.01 (1H, m, *CH_2_O-alkyl*), 3.76 (1H, m, *CH_2_O-alkyl glycerol*), 3.40–3.30 (4H, m, *CH_2_OC* and *CH_2_O-glycerol*), 1.70–1.50 (2H, m, *CH_2_CH_2_O-glycerol*), 1.48 (3H, s, *CH_3_ acetal*), 1.41 (3H, s, *CH_3_ acetal*), 1.29 (26H, m, *CH_2_*), 0.91 (3H, t, *J* = 6.8 Hz, *CH_3_*). ^13^C NMR-CDCl3, δ (ppm) 109.6, 74.2, 71.8, 71.3, 67.3, 31.7, 29.7, 29.2, 29.0, 26.6, 25.7, 23.2, 14.0.

**4-(*n*-Hexadec-7-enyloxymethyl)-2,2-dimethyl-1,3-dioxolane (3c).** This product was obtained from 1-hexadec-7-enol (0.5 g, 2.1 mmol). TLC performed in chloroform/acetone (90:10). Rf = 0.68. Yield = 76% (0.56 g). ^1^H NMR-CDCl3_,_ δ (ppm) 5.35 (2H, m, C*H*=C*H*), 4.26 (1H, m, *CH-glycerol*), 4.01 (1H, m, *CH_2_O-alkyl*), 3.76 (1H, m, *CH_2_O-alkyl glycerol*), 3.40–3.30 (4H, m, *CH_2_OC* and *CH_2_O-glycerol*), 2.03 (4H, m, ***CH_2_****-CH=CH-**CH_2_***), 1.75–1.56 (2H, m, *CH_2_CH_2_O-glycerol*), 1.47 (3H, s, *CH_3_ acetal*), 1.40 (3H, s, *CH_3_ acetal*), 1.29 (18H, m, *CH_2_*), 0.91 (3H, t, *J* = 7.0 Hz, *CH_3_*). ^13^C NMR-CDCl3_,_ δ (ppm) 130.5, 109.6, 74.2, 71.8, 71.3, 67.3, 31.7, 29.7, 29.3, 29.2, 29.0, 28.8, 28.0, 26.6, 25.7, 23.2, 14.0.

**4-(*n*-Heptadecyloxymethyl)-2,2-dimethyl-1,3-dioxolane (3d).** This product was obtained from 1-heptadecanol (0.5 g, 1.9 mmol). TLC performed in chloroform/acetone (90:10). Rf = 0.68. Yield = 60% (0.43 g). ^1^H NMR-CDCl3_,_ δ (ppm) 4.26 (1H, m, *CH-glycerol*), 4.01 (1H, m, *CH_2_O-alkyl*), 3.76 (1H, m, *CH_2_O-alkyl glycerol*), 3.40–3.30 (4H, m, *CH_2_OC* and *CH_2_O-glycerol*), 1.70–1.50 (2H, m, *CH_2_CH_2_O-glycerol*), 1.48 (3H, s, *CH_3_ acetal*), 1.41 (3H, s, *CH_3_ acetal*), 1.29 (28H, m, *CH_2_*), 0.91 (3H, t, *J* = 6.8 Hz, *CH_3_*). ^13^C NMR-CDCl3, δ (ppm) 109.6, 74.2, 71.8, 71.3, 67.3, 31.7, 29.7, 29.2, 29.0, 26.6, 25.7, 23.2, 14.0.

**4-(*n*-Octadecyloxymethyl)-2,2-dimethyl-1,3-dioxolane (3e).** This product was obtained from 1-octadecanol (0.5 g, 1.8 mmol). TLC performed in chloroform/acetone (90:10). Rf = 0.68. Yield = 65% (0.46 g). ^1^H NMR-CDCl3_,_ δ (ppm) 4.26 (1H, m, *CH-glycerol*), 4.01 (1H, m, *CH_2_O-alkyl*), 3.76 (1H, m, *CH_2_O-alkyl glycerol*), 3.40-3.30 (4H, m, *CH_2_OC* and *CH_2_O-glycerol*), 1.70–1.50 (2H, m, *CH_2_CH_2_O-glycerol*), 1.48 (3H, s, *CH_3_ acetal*), 1.41 (3H, s, *CH_3_ acetal*), 1.29 (30H, m, *CH_2_*), 0.91 (3H, t, *J* = 6,8 Hz, *CH_3_*). ^13^C NMR-CDCl3_,_ δ (ppm) 109.6, 74.2, 71.8, 71.3, 67.3, 31.7, 29.7, 29.2, 29.0, 26.6, 25.7, 23.2, 14.0.

**4-(*n*-Octadec-9-enyloxymethyl)-2,2-dimethyl-1,3-dioxolane (3f).** This product was obtained from 1-octadec-7-enol (0.5 g, 1.9 mmol). TLC performed in chloroform/acetone (90:10). Rf = 0.68. Yield = 78% (0.56 g). ^1^H NMR-CDCl3_,_ δ (ppm) 5.35 (2H, m, *CH=CH*), 4.26 (1H, m, *CH-glycerol*), 4.01 (1H, m, *CH_2_O-alkyl*), 3.76 (1H, m, *CH_2_O-alkyl glycerol*), 3.40–3.30 (4H, m, *CH_2_OC* and *CH_2_O-glycerol*), 2.03 (4H, m, ***CH_2_****-CH=CH-**CH_2_***), 1.75–1.56 (2H, m, *CH_2_CH_2_O-glycerol*), 1.47 (3H, s, *CH_3_ acetal*), 1.40 (3H, s, *CH_3_ acetal*), 1.29 (22H, m, *CH_2_*), 0.91 (3H, t, *J* = 7 Hz, *CH_3_*). ^13^C NMR-CDCl3_,_ δ (ppm) 130.5, 109.6, 74.2, 71.8, 71.3, 67.3, 31.7, 29.7, 29.3, 29.2, 29.0, 28.8, 28.0, 26.6, 25.7, 23.2, 14.0.

**4-(*n*-Nonadecyloxymethyl)-2,2-dimethyl-1,3-dioxolane (3g).** This product was obtained from 1-nonadecanol (0.5 g, 1.75 mmol). TLC performed in chloroform/acetone (90:10). Rf = 0.69. Yield = 70% (0.49 g). ^1^H NMR-CDCl3_,_ δ (ppm) 4.26 (1H, m, *CH-glycerol*), 4.01 (1H, m, *CH_2_O-alkyl*), 3.76 (1H, m, *CH_2_O*-*alkyl glycerol*), 3.40–3.30 (4H, m, *CH_2_OC* and *CH_2_O*-*glycerol*), 1.70–1.50 (2H, m, *CH_2_CH_2_O*-*glycerol*), 1.48 (3H, s, *CH_3_ acetal*), 1.41 (3H, s, *CH_3_ acetal*); 1.29 (32H, m, *CH_2_*), 0.91 (3H, t, *J* = 6.8 Hz, *CH_3_*). ^13^C NMR-CDCl3_,_ δ (ppm) 109.6, 74.2, 71.8, 71.3, 67.3, 31.7, 29.7, 29.2, 29.0, 26.6, 25.7, 23.2, 14.0.

**4-(*n*-Eicosyloxymethyl)-2,2-dimethyl-1,3-dioxolane (3h).** This product was obtained from 1-eicosanol (0.5 g, 1.7 mmol). TLC performed in chloroform/acetone (90:10). Rf = 0.68. Yield = 68% (0.47 g). ^1^H NMR-CDCl_3,_ δ (ppm) 4.26 (1H, m, *CH*-*glycerol*), 4.01 (1H, m, *CH_2_O*-*alkyl*), 3.76 (1H, m, *CH_2_O*-*alkyl glycerol*), 3.40–3.30 (4H, m, *CH_2_OC* and *CH_2_O*-*glycerol*), 1.70–1.50 (2H, m, *CH_2_CH_2_O*-*glycerol*), 1.48 (3H, s, *CH_3_ acetal*), 1.41 (3H, s, *CH_3_ acetal*), 1.29 (34H, m, *CH_2_*), 0.91 (3H, t, *J* = 6,8 Hz, *CH_3_*). ^13^C NMR-CDCl_3,_ δ (ppm) 109.6, 74.2, 71.8, 71.3, 67.3, 31.7, 29.7, 29.2, 29.0, 26.6, 25.7, 23.2, 14.0.

**4-(*n*-Docosyloxymethyl)-2,2-dimethyl-1,3-dioxolane (3i).** This product was obtained from 1-docosanol (0.5 g, 1.5 mmol). TLC performed in chloroform/acetone (90:10). Rf = 0.68. Yield = 79% (0.53 g). ^1^H NMR-CDCl3_,_ δ (ppm) 4.26 (1H, m, *CH-glycerol*), 4.01 (1H, m, *CH_2_O-alkyl*), 3.76 (1H, m, *CH_2_O-alkyl glycerol*), 3.40–3.30 (4H, m, *CH_2_OC* and *CH_2_O-glycerol*), 1.70–1.50 (2H, m, *CH_2_CH_2_O*-*glycerol*), 1.48 (3H, s, *CH_3_ acetal*), 1.41 (3H, s, *CH_3_ acetal*), 1.29 (38H, m, *CH_2_*), 0.91 (3H, t, *J* = 6.8 Hz, *CH_3_*). ^13^C NMR-CDCl3_,_ δ (ppm) 109.6, 74.2, 71.8, 71.3, 67.3, 31.7, 29.7, 29.2, 29.0, 26.6, 25.7, 23.2, 14.0.

#### 3.4.3. Preparation of the Ether-Glycerol 4a-i by Hydrolysis of Acetal **3a**–**i**

To a solution of 4-alkoxymethyl-2,2-dimethyl-1, 3-dioxolane (1.0 eq.) in methanol (approx. 5 mL), HCl (2 M, 2.0 eq.) was added and the solution was heated to reflux for 4 h. After cooling to room temperature, the mixture was poured into water, the organic layers were extracted with ether, dried over Na_2_SO_4_ and the solvents were removed under vacuum. The residue was purified by flash chromatography with hexane/ethyl acetate 3/1 *v/v*.

**3-(*n*-Tetradecyloxy)propane-1,2-diol (4a).** This product was obtained from compound **3a** (0.52 g, 1.58 mmol). TLC performed in hexane/ethyl acetate (75:25). Rf = 0.10. Yield = 98% (0.45 g). ^1^H NMR-CDCl3_,_ δ (ppm) 3.90 (1H, m, *CH-glycerol*), 3.80–3.71 (2H, m, *CH_2_O-alkyl*), 3.70–3.45 (4H, m, *CH_2_OH* and *CH_2_O-glycerol*), 2.62 (2H, bs, *2xOH*), 1.62 (2H, m, *CH_2_O-glycerol*),1.29 (22H, m, *CH_2_*), 0.91 (3H, t, *J* = 7 Hz, *CH_3_*). ^13^C NMR-CDCl3_,_ δ (ppm) 72.1, 71.8, 71.0, 63.5, 31.7, 29.7, 29.2, 29.0, 26.6, 23.2, 14.

**3-(*n*-Hexadecyloxy)propane-1,2-diol (4b).** This product was obtained from compound **3b** (0.71 g, 1.99 mmol). TLC performed in hexane/ethyl acetate (75:25). Rf = 0.10. Yield = 92% (0.58 g). ^1^H NMR-CDCl3_,_ δ (ppm) 3.90 (1H, m, *CH-glycerol*), 3.80–3.71 (2H, m, *CH_2_O-alkyl*), 3.70–3.45 (4H, m, *CH_2_OH* and *CH_2_O-glycerol*), 2.62 (2H, bs, *2xOH*), 1.62 (2H, m, *CH_2_O-glycerol*), 1.29 (26H, m, *CH_2_*), 0.91 (3H, t, *J* = 7 Hz, *CH_3_*). ^13^C NMR-CDCl3_,_ δ (ppm) 72.1, 71.8, 71.0, 63.5, 31.7, 29.7, 29.2, 29.0, 26.6, 23.2, 14.

**(7*E*)-3-(*n*-Hexadec-7-enyloxy)propane-1,2-diol (4c).** This product was obtained from compound **3c** (0.56 g, 1.58 mmol). TLC performed in hexane/ethyl acetate (75:25). Rf = 0.10. Yield = 98% (0.49 g). ^1^H NMR-CDCl_3,_ δ (ppm) 5.35–5.30 (2H, m, *CH=CH*), 3.90 (1H, m, *CH-glycerol*), 3.82–3.75 (2H, m, *CH_2_O-alkyl*), 3.69–3.47 (4H, m, *CH_2_OH* and *CH_2_O-glycerol*), 2.60 (2H, bs, *2xOH*), 2.03 (4H, m, ***CH_2_****-CH=CH-**CH_2_***), 1.63 (2H, m, *CH_2_O-glycerol*), 1.28 (18H, m, *CH_2_*), 0.91 (3H, t, *J* = 6.8 Hz, *CH_3_*). ^13^C NMR-CDCl_3,_ δ (ppm) 130.5, 72.1, 71.8, 71.0, 63.5, 31.7, 29.7, 29.3, 29.2, 29.0, 28.8, 28.0, 26.6, 23.2, 14.0.; HRMS ([M+H]^+^) calcd for [C_19_H_38_O_3_H]^+^ 315.2894, found 315.2894.

**3-(*n*-Heptadecyloxy)propane-1,2-diol (4d).** This product was obtained from compound **3d** (0.43 g, 1.16 mmol). TLC performed in hexane/ethyl acetate (75:25). Rf = 0.10. Yield = 95% (0.36 g). ^1^H NMR-CDCl3_,_ δ (ppm) 3.90 (1H, m, *CH-glycerol*), 3.80–3.71 (2H, m, *CH_2_O-alkyl*), 3.70–3.45 (4H, m, *CH_2_OH* and *CH_2_O-glycerol*), 2.62 (2H, bs, *2xOH*), 1.62 (2H, m, *CH_2_O-glycerol*), 1.29 (28H, m, *CH_2_*), 0.91 (3H, t, *J* = 7 Hz, C*H*_3_). ^13^C NMR-CDCl3_,_ δ (ppm) 72.1, 71.8, 71.0, 63.5, 31.7, 29.7, 29.2, 29.0, 26.6, 23.2, 14.; HRMS ([M+H]^+^) calcd for [C_20_H_42_O_3_H]^+^ 331.3207, found 331.3210.

**3-(*n*-Octadecyloxy)propane-1,2-diol (4e).** This product was obtained from compound **3e** (0.46 g, 1.20 mmol). TLC performed in hexane/ethyl acetate (75:25). Rf = 0.10. Yield = 96% (0.40 g). ^1^H NMR-CDCl3_,_ δ (ppm) 3.90 (1H, m, *CH-glycerol*), 3.80–3.71 (2H, m, *CH_2_O-alkyl*), 3.70–3.45 (4H, m, *CH_2_OH* and *CH_2_O-glycerol*), 2.62 (2H, bs, *2xOH*), 1.62 (2H, m, *CH_2_O-glycerol*), 1.29 (30H, m, *CH_2_*), 0.91 (3H, t, *J* = 7 Hz, *CH_3_*). ^13^C NMR-CDCl3, δ (ppm) 72.1, 71.8, 71.0, 63.5, 31.7, 29.7, 29.2, 29.0, 26.6, 23.2, 14.

**(9*E*)-3-(*n*-Octadec-9-enyloxy)propane-1,2-diol (4f).** This product was obtained from compound **3f** (0.56 g, 1.46 mmol). TLC performed in hexane/ethyl acetate (75:25). Rf = 0.10. Yield = 96% (0.48 g). ^1^H NMR-CDCl3, δ (ppm) 5.37–5.30 (m, 2H, *CH=CH*), 3.88–3.83 (m, 1H, *CH*), 3.72 (ddd, *J* = 11.1, 7.0, 3.9 Hz, 1H, *CH*), 3.68–3.62 (m, 1H, *CH*), 3.54 (dd, *J* = 9.7, 3.8 Hz, 1H, *CH*), 3.50 (dd, *J* = 9.8, 6.0 Hz, 1H, *CH*), 3.48 (dd, *J* = 6.4, 9.2 Hz, 1H, *CH*), 3.45 (dd, *J* = 9.2, 6.5 Hz, 1H, *CH*), 2.58 (d, *J* = 5.1 Hz, 1H, *CH*), 2.15 (m, 1H, *CH*), 2.01 (dd, *J* = 12.8, 6.6 Hz, 4H, ***CH_2_****-CH=CH-**CH_2_***), 1.39–1.15 (m, 24H, *CH_2_*), 0.88 (t, *J* = 6.9 Hz, 3H, *CH_3_*). ^13^C NMR-CDCl3, δ (ppm), 130.5, 72.1, 71.8, 71.0, 63.5, 31.7, 29.7, 29.3, 29.2, 29.0, 28.8, 28.0, 26.6, 23.2, 14.0.

**3-(*n*-Nonadecyloxy)propane-1,2-diol (4g).** This product was obtained from compound **3g** (0.49 g, 1.23 mmol). TLC performed in hexane/ethyl acetate (75:25). Rf = 0.10. Yield = 95% (0.42 g). ^1^H NMR-CDCl3_,_ δ (ppm) 3.90 (1H, m, *CH-glycerol*), 3.80–3.71 (2H, m, *CH_2_O-alkyl*), 3.70–3.45 (4H, m, *CH_2_OH* and *CH_2_O-glycerol*), 2.62 (2H, bs, *2xOH*), 1.62 (2H, m, *CH_2_O-glycerol*), 1.29 (32H, m, *CH_2_*), 0.91 (3H, t, *J* = 7 Hz, *CH_3_*). ^13^C NMR-CDCl3_,_ δ (ppm) 72.1, 71.8, 71.0, 63.5, 31.7, 29.7, 29.2, 29.0, 26.6, 23.2, 14.; HRMS ([M+H]^+^) calcd for [C_22_H_46_O_3_H]^+^ 359.3520, found 359.3523.

**3-(*n*-Eicosyloxy)propane-1,2-diol (4h).** This product was obtained from compound **3h** (0.47 g, 1.14 mmol). TLC performed in hexane/ethyl acetate (75:25). Rf = 0.10. Yield = 95% (0.40 g). ^1^H NMR-CDCl3, δ (ppm) 3.90 (1H, m, *CH-glycerol*), 3.80–3.71 (2H, m, *CH_2_O-alkyl*), 3.70–3.45 (4H, m, *CH_2_OH* and *CH_2_O-glycerol*), 2.62 (2H, bs, *2xOH*), 1.62 (2H, m, *CH_2_O-glycerol*), 1.29 (34H, m, *CH_2_*), 0.91 (3H, t, *J* = 7 Hz, *CH_3_*). ^13^C NMR-CDCl3_,_ δ (ppm) 72.1, 71.8, 71.0, 63.5, 31.7, 29.7, 29.2, 29.0, 26.6, 23.2, 14.

**3-(*n*-Docosyloxy)propane-1,2-diol (4i).** This product was obtained from compound **3i** (0.53 g, 1.20 mmol). TLC performed in hexane/ethyl acetate (75:25). Rf = 0.10. Yield = 95% (0.46 g). ^1^H NMR-CDCl3_,_ δ (ppm) 3.90 (1H, m, *CH-glycerol*), 3.80–3.71 (2H, m, *CH_2_O-alkyl*), 3.70–3.45 (4H, m, *CH_2_OH* and *CH_2_O-glycerol*), 2.62 (2H, bs, *2xOH*), 1.62 (2H, m, *CH_2_O-glycerol*), 1.29 (38H, m, *CH_2_*), 0.91 (3H, t, *J* = 7 Hz, *CH_3_*). ^13^C NMR-CDCl3_,_ δ (ppm) 72.1, 71.8, 71.0, 63.5, 31.7, 29.7, 29.2, 29.0, 26.6, 23.2, 14.

#### 3.4.4. Derivation Procedure: Preparation of 3-(Alkoxy)propane-1,2-diacetate 

Method A [43]: To a solution of 3-(alkoxy)propane-1,2-diol (1.0 eq.) in pyridine (4.0 eq.), acetic anhydride (5.5 eq.) was added and the solution was heated to 60 °C for 30 min and stirred at room temperature overnight. The organic layer was extracted with diethyl ether, washed with HCl (1 M), an aqueous solution of K_2_CO_3_ (10%), and a saturated solution of NaCl. The resulting organic layer was dried over Na_2_SO_4_ and the solvents were removed under vacuum. The residue was purified by flash chromatography using chloroform/acetone (90:10).

Method B [44]: In a screw cap tube, to the 3-(alkoxy) propane-1,2-diol (1.0 eq.), acetyl chloride (2.1 eq.) was added. The tube was closed under inert gas and heated at 140 °C for 5 min. After cooling at room temperature, diethyl ether was added. The excess of acetyl chloride was neutralized by dropwise addition of a saturated aqueous solution of sodium hydrogen carbonate until no more bubbles were observed. The organic layer was filtered over Na_2_SO_4_ and evaporated to dryness to give the pure compound as a white powder.

**3-(Tetradecyloxy)propane-1,2-diacetate (5a).** This product was obtained from compound **4a** by using method A (0.45 g, 1.56 mmol). TLC performed in chloroform/acetone (96:4). Rf = 0.77. Yield = 95% (0.55 g). ^1^H NMR-CDCl3_,_ δ (ppm) 5.20 (1H, m, *CH-glycerol*), 4.50 & 4.07 (2H, m, *CH_2_O-acetate*), 3.67 & 3.46 (2H, m, *CH_2_O-alkyl*), 3.33–3.30 (2H, m, *CH_2_O-glycerol*), 2.07 (3H, s, *CH_3_ acetate*), 2.03 (3H, s, *CH_3_ acetate*), 1.55–1.47 (4H, m, *CH_2_-alkyl*), 1.30–1.24 (20H, m, *CH_2_*), 0.89 (3H, t, *J* = 7 Hz, *CH_3_*). ^13^C NMR-CDCl3_,_ δ (ppm) 171.8, 170.5, 71.8, 70.6, 70.0, 63.7, 31.7, 29.7, 29.2, 29.0, 26.6, 23.2, 21.3, 20.7, 14.

**3-(Hexadecyloxy)propane-1,2-diacetate (5b).** This product was obtained from compound **4b** by using method A (0.58 g, 1.83 mmol). TLC performed in chloroform/acetone (96:4). Rf = 0.77. Yield = 95% (0.70 g). ^1^H NMR-CDCl3_,_ δ (ppm) 5.20 (1H, m, *CH-glycerol*), 4.49 & 4.09 (2H, m, *CH_2_O-acetate*), 3.67 & 3.46 (2H, m, *CH_2_O-alkyl*), 3.34–3.30 (2H, m, *CH_2_O-glycerol*), 2.07 (3H, s, *CH_3_-acetate*), 2.03 (3H, s, *CH_3_-acetate*), 1.55–1.47 (4H, m, *CH_2_-alkyl*), 1.30–1.24 (24H, m, *CH_2_*), 0.89 (3H, t, *J* = 7 Hz, *CH_3_*). ^13^C NMR-CDCl3_,_ δ (ppm) 171.8, 170.5, 71.8, 70.6, 70.0, 63.7, 31.7, 29.7, 29.2, 29.0, 26.6, 23.2, 21.3, 20.7, 14.

**(*E*)-3-(Hexadec-7-enyloxy)propane-1,2-diacetate (5c).** This product was obtained from compound **4c** by using method B (0.49 g, 1.56 mmol). TLC performed in chloroform/acetone (96:4). Rf = 0.77. Yield = 98% (0.61 g). ^1^H NMR-CDCl3_,_ δ (ppm) 5.34-5.30 (2H, m, *CH=CH*), 5.20 (1H, m, *CH-glycerol*), 4.50 (1H, m, *CH_2_O-acetate*), 4.04 (1H, m, *CH_2_O-acetate*), 3.66 (1H, m, *CH_2_O-alkyl*), 3.44 (H, m, *CH_2_O-alkyl*), 3.33 (2H, m, *CH_2_O-glycerol*), 2.07–1.98 (10H, m, *2 x CH_3_ acetate* and ***CH_2_****_-_CH=CH-**CH_2_***), 1.55 (2H, m, *CH_2_CH_2_O*), 1.47 (2H, m, *CH_2_*), 1.33–1.26 (16H, m, *CH_2_*), 0.89 (3H, t, *J* = 7 Hz, *CH_3_*)_._^13^C NMR-CDCl3_,_ δ (ppm) 171.8, 170.5, 130.5, 71.8, 70.6, 70.0, 63.7, 31.7, 29.7, 29.3, 29.2, 29.0, 28.8, 28.0, 26.6, 23.2, 21.3, 20.7, 14.0.

**3-(Heptadecyloxy)propane-1,2-diacetate (5d).** This product was obtained from compound **4d** by using method A (0.36 g, 1.08 mmol). TLC performed in chloroform/acetone (96:4). Rf = 0.77. Yield = 92% (0.42 g). ^1^H NMR-CDCl3_,_ δ (ppm) 5.20 (1H, m, *CH-glycerol*), 4.49 & 4.09 (2H, m, *CH_2_O-acetate*), 3.67 & 3.46 (2H, s, *CH_2_O-alkyl*), 3.34–3.30 (2H, m, *CH_2_O-glycerol*), 2.07 (3H, s, *CH_3_-acetate*), 2.03 (3H, s, *CH_3_-acetate*), 1.55–1.47 (4H, m, *CH_2_-alkyl*), 1.30–1.24 (26H, m, *CH_2_*), 0.89 (3H, t, *J* = 7 Hz, *CH_3_*). ^13^C NMR-CDCl3_,_ δ (ppm) 171.8, 170.5, 71.8, 70.6, 70.0, 63.7, 31.7, 29.7, 29.2, 29.0, 26.6, 23.2, 21.3, 20.7, 14.

**3-(Octadecyloxy)propane-1,2-diacetate (5e).** This product was obtained from compound **4e** by using method B (0.40 g, 1.16 mmol). TLC performed in chloroform/acetone (96:4). Rf = 0.77. Yield = 93% (0.46 g). ^1^H NMR-CDCl3_,_ δ (ppm) 5.20 (1H, m, *CH-glycerol*), 4.49 & 4.09 (2H, d, *CH_2_O-acetate*), 3.67 & 3.46 (2H, m, *CH_2_O-alkyl*), 3.34–3.30 (2H, m, *CH_2_O-glycerol*), 2.07 (3H, s, *CH_3_-acetate*), 2.03 (3H, s, *CH_3_-acetate*), 1.55–1.47 (4H, m, *CH_2_-alkyl*), 1.30–1.24 (28H, m, *CH_2_*), 0.89 (3H, t, *J* = 7 Hz, *CH_3_*). ^13^C NMR-CDCl3_,_ δ (ppm) 171.8, 170.5, 71.8, 70.6, 70.0, 63.7, 31.7, 29.7, 29.2, 29.0, 26.6, 23.2, 21.3, 20.7, 14.

**(9*E*)-3-(Octadec-9-enyloxy)propane-1,2-diacetate (5f).** This product was obtained from compound **4f** by using method B (0.48 g, 1.40 mmol). TLC performed in chloroform/acetone (96:4). Rf = 0.77. Yield = 96% (0.57 g). ^1^H NMR-CDCl3_,_ δ (ppm) 5.38–5.31 (m, 2H, *CH=CH*), 5.21–5.16 (m, 1H, *CH*), 4.33 (dd, *J* = 12.0, 3.6 Hz, 1H, *CH*), 4.16 (dd, *J* = 12.0, 6.4 Hz, 1H, *CH*), 3.55 (dd, *J* = 10.8, 5.4 Hz, 1H, *CH*), 3.54 (dd, *J* = 10.6, 5.1 Hz, 1H, *CH*), 3.50–3.38 (m, 2H, *CH_2_*), 2.09 (s, 3H, CH_3_ acetate), 2.07 (s, 3H, *CH_3_ acetate*), 2.01 (dd, *J* = 12.8, 6.8 Hz, 4H, ***CH_2_****-CH=CH-**CH_2_***), 1.39–1.20 (m, 24H, *CH2*), 0.88 (t, *J* = 7.0 Hz, 3H, *CH_3_*). ^13^C NMR-CDCl3_,_ δ (ppm) 171.8, 170.5, 130.5, 71.8, 70.6, 70.0, 63.7, 31.7, 29.7, 29.3, 29.2, 29.0, 28.8, 28.0, 26.6, 23.2, 21.3, 20.7, 14.0.

**3-(Nonadecyloxy)propane-1,2-diacetate (5g).** This product was obtained from compound **4g** by using method A (0.42 g, 1.17 mmol). TLC performed in chloroform/acetone (96:4). Rf = 0.77. Yield = 95% (0.49 g). ^1^H NMR-CDCl3_,_ δ (ppm) 5.20 (1H, m, *CH-glycerol*), 4.49 & 4.09 (2H, m, *CH_2_O-acetate*), 3.67 & 3.46 (2H, m, *CH_2_O-alkyl*), 3.34–3.30 (2H, m, *CH_2_O-glycerol*), 2.07 (3H, s, *CH_3_-acetate*), 2.03 (3H, s, *CH_3_-acetate*), 1.55–1.47 (4H, m, *CH_2_-alkyl*), 1.30–1.24 (30H, m, *CH_2_*), 0.89 (3H, t, *J* = 7 Hz, *CH_3_*). ^13^C NMR-CDCl3_,_ δ (ppm) 171.8, 170.5, 71.8, 70.6, 70.0, 63.7, 31.7, 29.7, 29.2, 29.0, 26.6, 23.2, 21.3, 20.7, 14.

**3-(Eicosyloxy)propane-1,2-diacetate (5h).** This product was obtained from compound **4h** by using method B (0.40 g, 1.07 mmol). TLC performed in chloroform/acetone (96:4). Rf = 0.77. Yield = 95% (0.47 g). ^1^H NMR-CDCl3_,_ δ (ppm) 5.16–5.21 (1H, m, *CH-glycerol*), 4.33 & 4.15 (2H, d, *CH_2_O-acetate*), 3.53 & 3.56 (2H, m, *CH_2_O-alkyl*), 3.38–3.48 (2H, m, *CH_2_O-glycerol*), 2.09 (3H, s, *CH_3_-acetate*), 2.07 (3H, s, *CH_3_-acetate*), 1.61–1.50 (H, m, *CH_2_-alkyl*), 1.35–1.25 (34H, m, *CH_2_*), 0.87–0.89 (3H, t, *J* = 7 Hz, *CH_3_*). ^13^C NMR-CDCl3_,_ δ (ppm) 171.8, 170.5, 71.8, 70.6, 70.0, 63.7, 31.7, 29.7, 29.2, 29.0, 26.6, 23.2, 21.3, 20.7, 14.

**3-(Docosyloxy)propane-1,2-diacetate (5i).** This product was obtained from compound **4i** by using method A (0.46 g, 1.15 mmol). TLC performed in chloroform/acetone (96:4). Rf = 0.77. Yield = 95% (0.53 g). ^1^H NMR-CDCl_3,_ δ (ppm) 5.18 (m, 1H, C*H*-glycerol), 4.33 (dd, *J* = 12.0, 3.6 Hz, 1H, C*H*_2_O-acetate), 4.16 (dd, *J* = 12.0, 6.4 Hz, 1H, C*H*_2_O-acetate), 3.55 (dd, *J* = 10.8, 5.4 Hz, 1H, C*H*_2_O-alkyl), 3.52 (dd, *J* = 10.7, 5.3 Hz, 1H, C*H*_2_O-alkyl), 3.39–3.48 (2H, m, C*H*_2_O-glycerol), 2.07 (3H, s, C*H*_3_-acetate), 2.03 (3H, s, C*H*_3_-acetate), 1.55–1.47 (4H, m, C*H*_2_-alkyl), 1.30–1.24 (36H, m, C*H*_2_), 0.89 (t, *J* = 6.9 Hz, 3H, C*H*_3_). ^13^C NMR-CDCl3_,_ δ (ppm) 171.1, 170.7, 71.8, 70.6, 70.0, 63.7, 31.7, 29.7, 29.2, 29.0, 26.6, 23.2, 21.3, 20.7, 14.

### 3.5. Analytical Chemistry

#### Analytical Biochemistry

The total lipids were extracted from 2 mg chimera and shark liver oil by Folch’s method [48]. Alkylglycerols were obtained by separation of lipid extracts by silica gel thin layer chromatography with chloroform/acetone (90:10, *v/v*) as the developing solvent. Separated lipids were visualized under ultraviolet light after spraying with 0.2% solution of 2,7-dichlorofluorescein in ethanol. Alkylglycerols were scraped into glass tubes with Teflon screw caps and acetylated with a large excess (600 µL) of acetyl chloride for 5 min at 140 °C. Then, the solution was neutralized with NaHCO_3_ and extracted with diethyl ether. After evaporation, the sample was taken up with hexane.

For the chromatography procedure, the samples were analyzed in batches of 13 samples comprising 10 oil samples, 3 samples comprising two blanks and one mixture of synthesized alkyl lipids serving as a reference. The alkylglycerols were identified by comparison of their retention times and their fragmentation peaks in mass spectroscopy with those of the synthesized standards (see Table 1). 

### 3.6. Validation of the Analytical Procedure

The Table 2 represents the Statistical data for analytical procedure for GC and show the fiability of the method used.

## 4. Conclusions

This work demonstrates that a series of 1-*O*-alkyl-glycerols featuring mainly saturated lipid chain lengths (14:0, 16:0, 17:0, 19:0, 20:0, 22:0) and two mono-unsaturated lipid chains (16:1, 18:1) were easily prepared and derivatized as diacetate compounds that were used as standards for GC analysis. These standards were used to determine the molecular species of AEL present in shark oils. It would be interesting to use these standards to also determine the AEL composition in tumors. Indeed, it is known that the ether-lipids fraction is larger in tumors than non-tumoral tissues, but the molecular species have not been precisely identified. Moreover, in a second time it would be interesting to synthesize standards that would identify alkenyl-lipids. Indeed, alkenyl-lipids were found to be highly present in the membrane phospholipids of some tissues such as the heart and brain [50]. The determination of the composition of the alkenyl-lipids in tumors will help to understand the role of these lipids in the biology of tumors cells.

## Figures and Tables

**Table 1:** Mass spectroscopy data.

Alkyl Chain R =	Retention Time (min)	Molecular Weight (g. × mol^−1^)	Most Characteristic Peaks (*m*/*z*, (%))
C_14_H_29_ (C14:0)	13.6	372.54	43 (base peak), 57 (60), 71 (35), 83 (30), 97 (26), 111 (13), 117 (19), 159 (119), 223 (4), 269 (6), 313 (35)
C_16_H_31_ (C16:1)	15.0	398.58	43 (base peak), 57 (63), 69 (44), 81 (75), 95(39), 117 (31), 159 (63), 222 (15), 281 (12), 339 (25)
C_16_H_33_ (C16:0)	15.7	400.59	43 (base peak), 57 (62), 71 (38), 83 (28), 97 (27), 111 (14), 159 (7), 219 (<5), 251 (5), 255 (<5), 297 (6), 341 (67)
C_17_H_35_ (C17:0)	16.8	414.62	43 (base peak), 57 (62), 83 (28), 97 (27), 111 (15), 117 (22) 159 (25), 209 (<5), 267 (8), 311 (12), 355 (54)
C_18_H_35_ (C18:1)	17.5	426.63	43 (base peak), 55 (52), 67 (48), 81(72), 95 (44), 117 (18), 159 (75), 191 (7), 219 (<5), 281 (12), 341 (7), 367 (25)
C_18_H_37_ (C18:0)	17.8	428.65	43 (base peak), 57 (70), 71(45), 85 (34), 97 (33), 111 (18), 117 (27), 159 (27), 191 (<5), 281 (10), 325 (9), 369 (37)
C_19_H_39_ (C19:0)	18.7	442.67	43 (base peak), 57 (65), 83 (38), 71 (38), 97 (36), 111 (20), 117(32), 159 (30), 233 (<5), 281 (7), 339 (6), 383 (47)
C_20_H_41_ (C20:0)	19.7	456.70	43 (base peak), 57 (70), 71 (42), 83 (36), 97 (36), 111 (18), 117 (32),159 (27), 125 (10), 207 (23), 295 (12), 355 (32), 398(40)
C_22_H_45_ (C22:0)	21.4	484.75	43 (base peak), 57 (70), 71 (38), 83 (28), 97 (38), 111 (22), 117 (12), 125 (12) 159 (30), 207 (22), 281 (8), 355 (10), 381 (6), 423 (27)

**Table 2:** Statistical data for analytical procedure for GC

Parameters Studied	Results
Selectivity	Correct
Fidelity	
Repeatability	Correct (CVr = 3.73%)
Reproducibility	Correct (CVR = 3.75%)
Linearity	Correct (no deviation from linearity, R^2^ = 0.992)
Detection limit LOD	2.9 pg (on-column injector, FID)
Quantification limit LOQ	17.5 pg (on-column injector, FID)
Stability	1 year at −80 °C

## Supplementary Materials

The following are available online at http://www.mdpi.com/1660-3397/16/4/101/s1. Figure SI1-1. ^1^H NMR (CDCl_3_) spectrum of compound **2**, Figure SI1-2. ^1^H NMR (CDCl_3_) spectrum of compound **3f**, Figure SI1-3. ^1^H NMR (CDCl_3_) spectrum of compound **4f**, Figure SI1-4. 2D NMR HSQC (CDCl_3_) spectrum of compound **4f**, Figure SI1-5. ^1^H NMR (CDCl_3_) spectrum of compound **5f**, Figure SI1-6. 2D NMR HSQC (CDCl_3_) spectrum of compound **5f**, Figure SI1-7. ^1^H NMR (CDCl_3_) spectrum of compound **5h**, Figure SI1-8. 2D NMR HSQC (CDCl_3_) spectrum of compound **5h**, Figure SI2-1. GC-MS chromatogram of a mixture of synthesized alkyl-glycerolipids and Figure SI2-2. Mass spectroscopy data.
