# Supplementary material for: Synthesis of Alkyl-Glycerolipids Standards for Gas Chromatography Analysis: Application for Chimera and Shark Liver Oils
Source: Mar Drugs. 2018 Mar 23;16(4):101. doi: 10.3390/md16040101 (PMC5923388; doi:10.3390/md16040101)
Supplement: Supplementary file 1 [file marinedrugs-16-00101-s001.pdf]

Supplementary Materials

Synthesis of Alkyl-Glycerolipid Standards for Gas Chromatography Analysis: Application for Chimera and Shark Liver Oils

Michelle Pinault[1], Cyrille Guimaraes[1], Hélène Couthon[2], Jérôme Thibonnet[3], Delphine Fontaine[1], Aurélie Chantôme[1,4], Stephan Chevalier[1,4], Pierre Besson[1,4], Paul-Alain Jaffrès[2*] and Christophe Vandier[1,5*]

Summary

1. Spectroscopic characterization of synthesized compounds 3

(2,2-dimethyl-1,3-dioxolan-4-yl)methyl 4-methylbenzenesulfonate 2 3

Figure SI1-1: ^{1}H NMR ($CDCl_3$) spectrum of compound **2** 3

4-(n-octadec-9-enyloxymethyl)-2,2-dimethyl-1,3-dioxolane 3f 4

Figure SI1-2 : ^{1}H NMR ($CDCl_3$) spectrum of compound **3f** 4

(E)-3-(octadec-9-enyloxy)propane-1,2-diol 4f 5

Figure SI1-3 : ^{1}H NMR ($CDCl_3$) spectrum of compound **4f** 5

Figure SI1-4 : 2D NMR HSQC ($CDCl_3$) spectrum of compound **4f** 6

(E)-3-(octadec-9-enyloxy)propane-1,2-diacetate 5f 7

Figure SI1-5 : ^{1}H NMR ($CDCl_3$) spectrum of compound **5f** 7

Figure SI1-6 : 2D NMR HSQC ($CDCl_3$) spectrum of compound **5f** 8

3-(eicosyloxy)propane-1,2-diacetate 5h 9

Figure SI1-7 : ^{1}H NMR ($CDCl_3$) spectrum of compound **5h** 10

Figure SI1-8 : 2D NMR HSQC ($CDCl_3$) spectrum of compound **5h** 11

2. Chromatographic characterization of synthesized compounds 11

GC-MS chromatogram of synthesized alkyl-glycerolipids 11

Figure SI1-9 : GC-MS chromatogram of a mixture of synthesized alkyl-glycerolipids 12

Figure SI1-10 : Mass spectroscopy data 12

1. Spectroscopic Characterization of Synthesized Compounds

(2,2-dimethyl-1,3-dioxolan-4-yl)methyl 4-methylbenzenesulfonate **2**

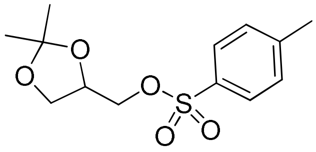

2

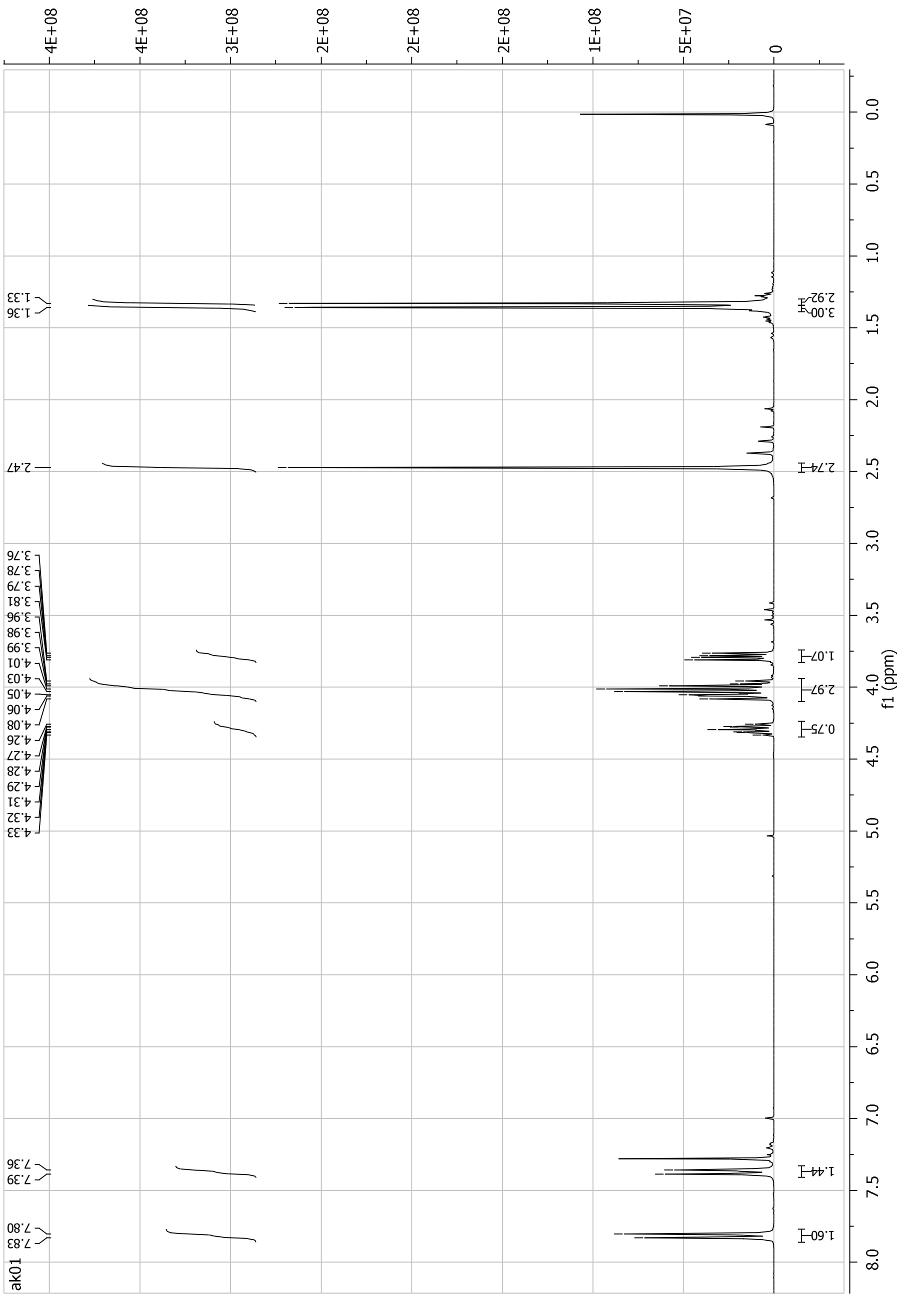

Figure SI1-1. ^{1}H NMR ($CDCl_3$) spectrum of compound **2.**

4-(n-octadec-9-enyloxymethyl)-2,2-dimethyl-1,3-dioxolane **3f**

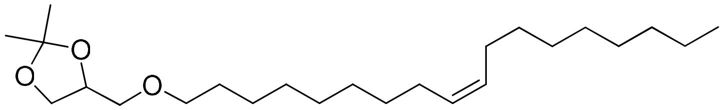

3f

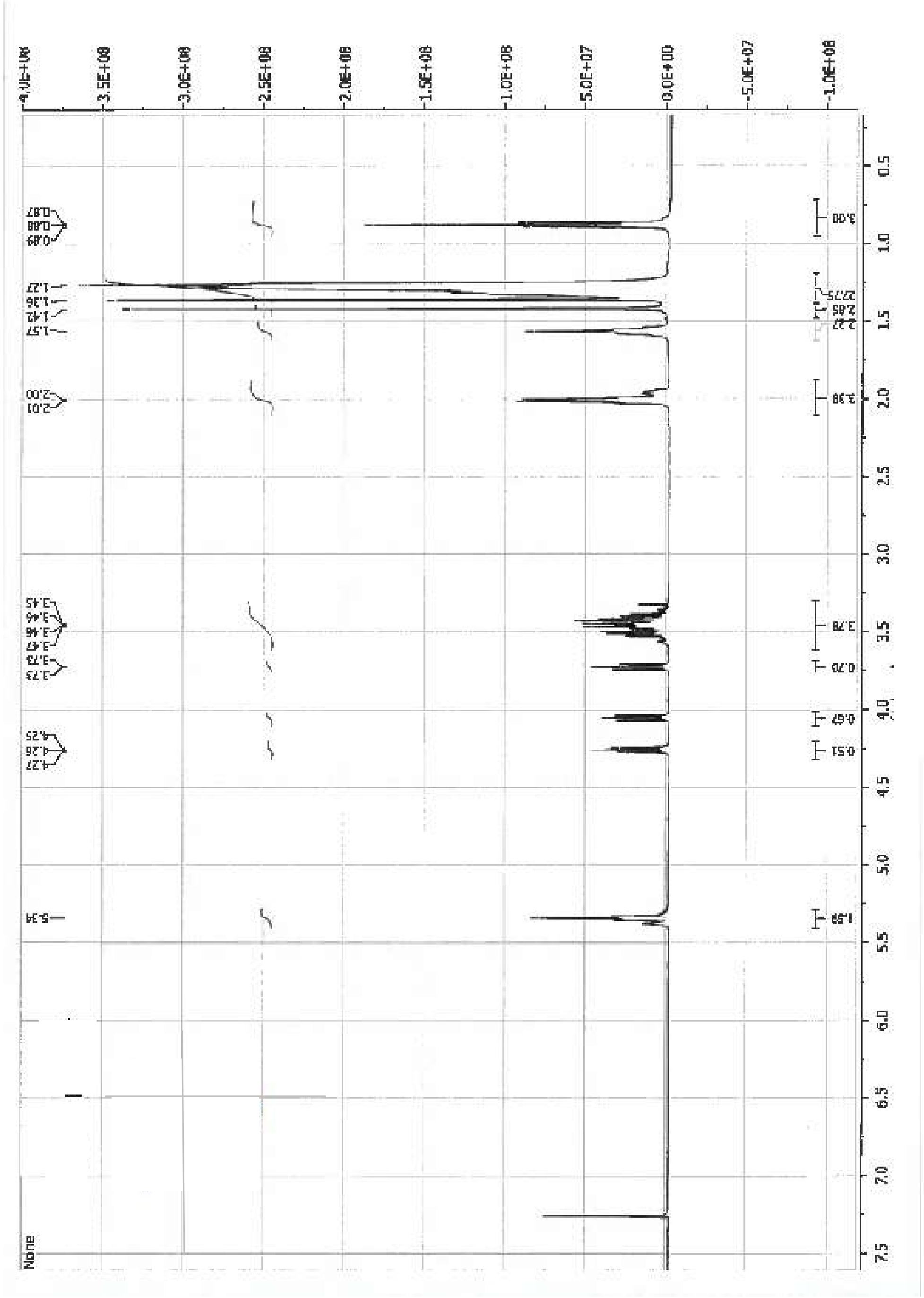

Figure SI1-2. ^{1}H NMR ($CDCl_3$) spectrum of compound **3f**.

(E)-3-(octadec-9-enyloxy)propane-1,2-diol 4f

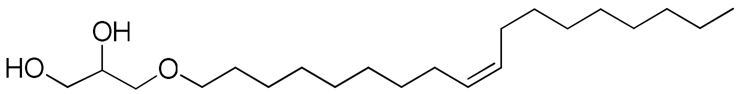

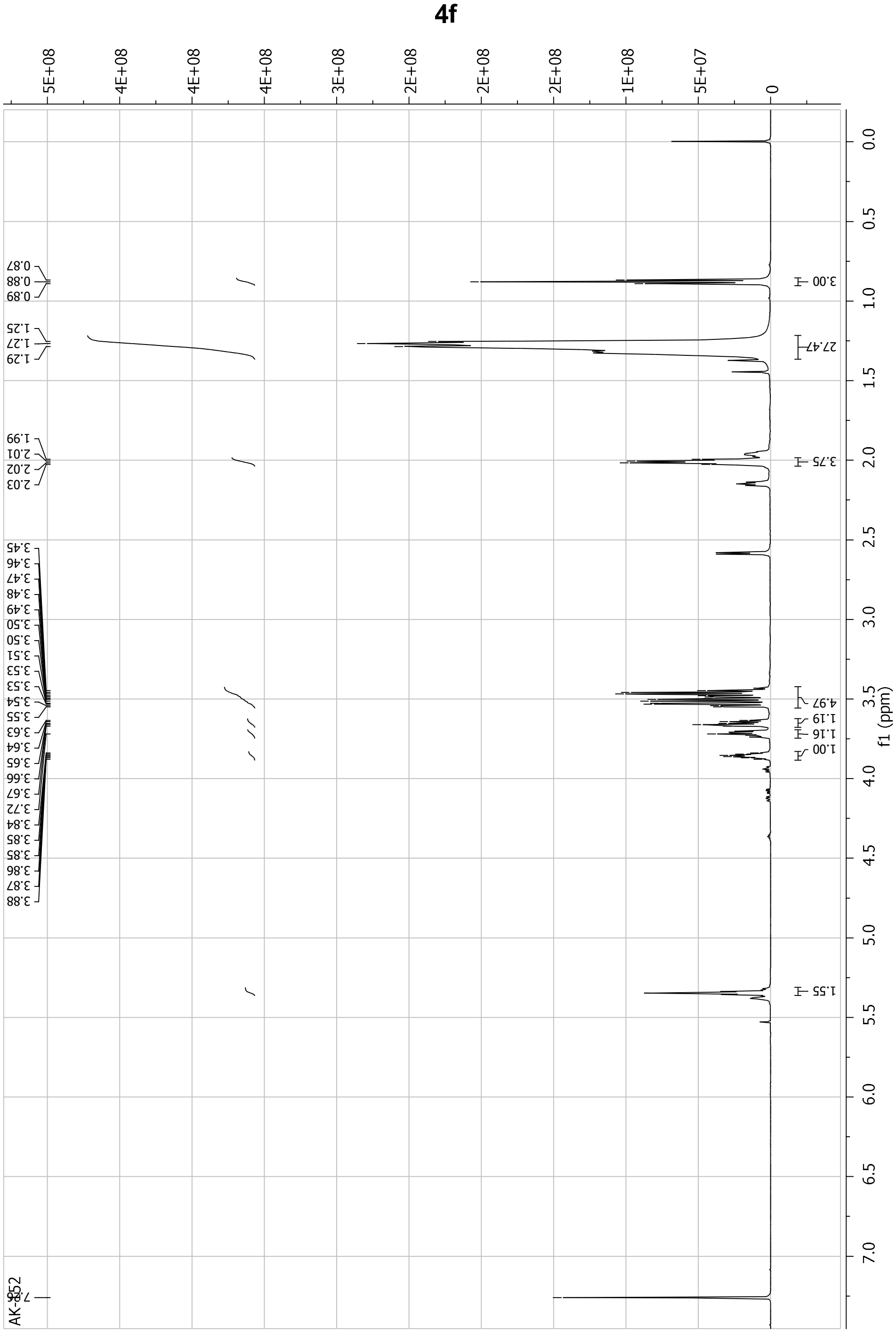

Figure SI1-3. ^{1}H NMR ($CDCl_3$) spectrum of compound **4f**.

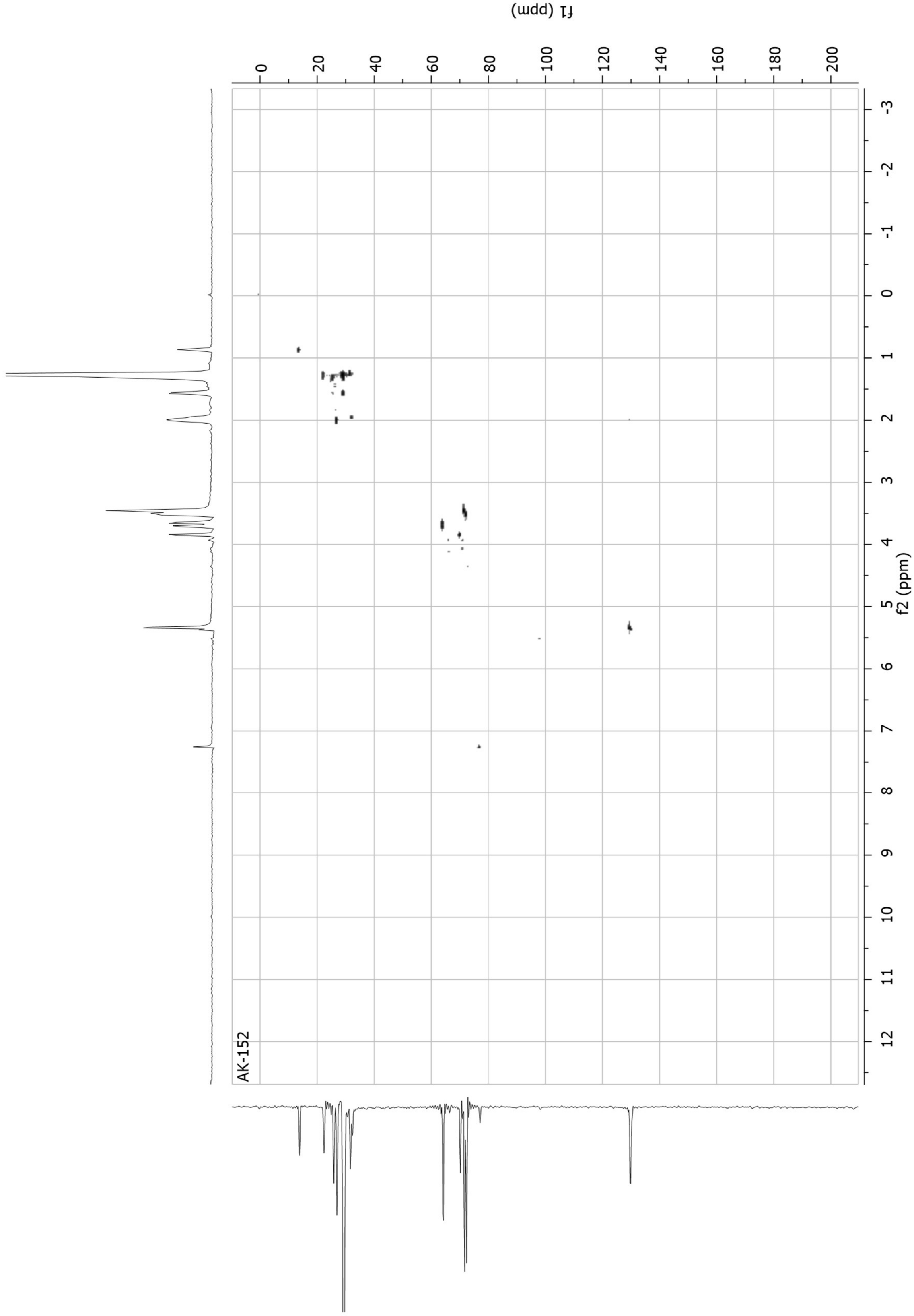

Figure SI1-4. 2D NMR HSQC ($CDCl_3$) spectrum of compound **4f**.

(E)-3-(octadec-9-enyloxy)propane-1,2-diacetate **5f**

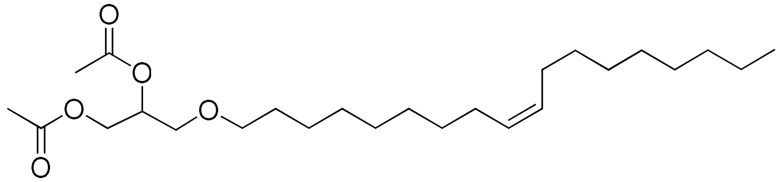

5f

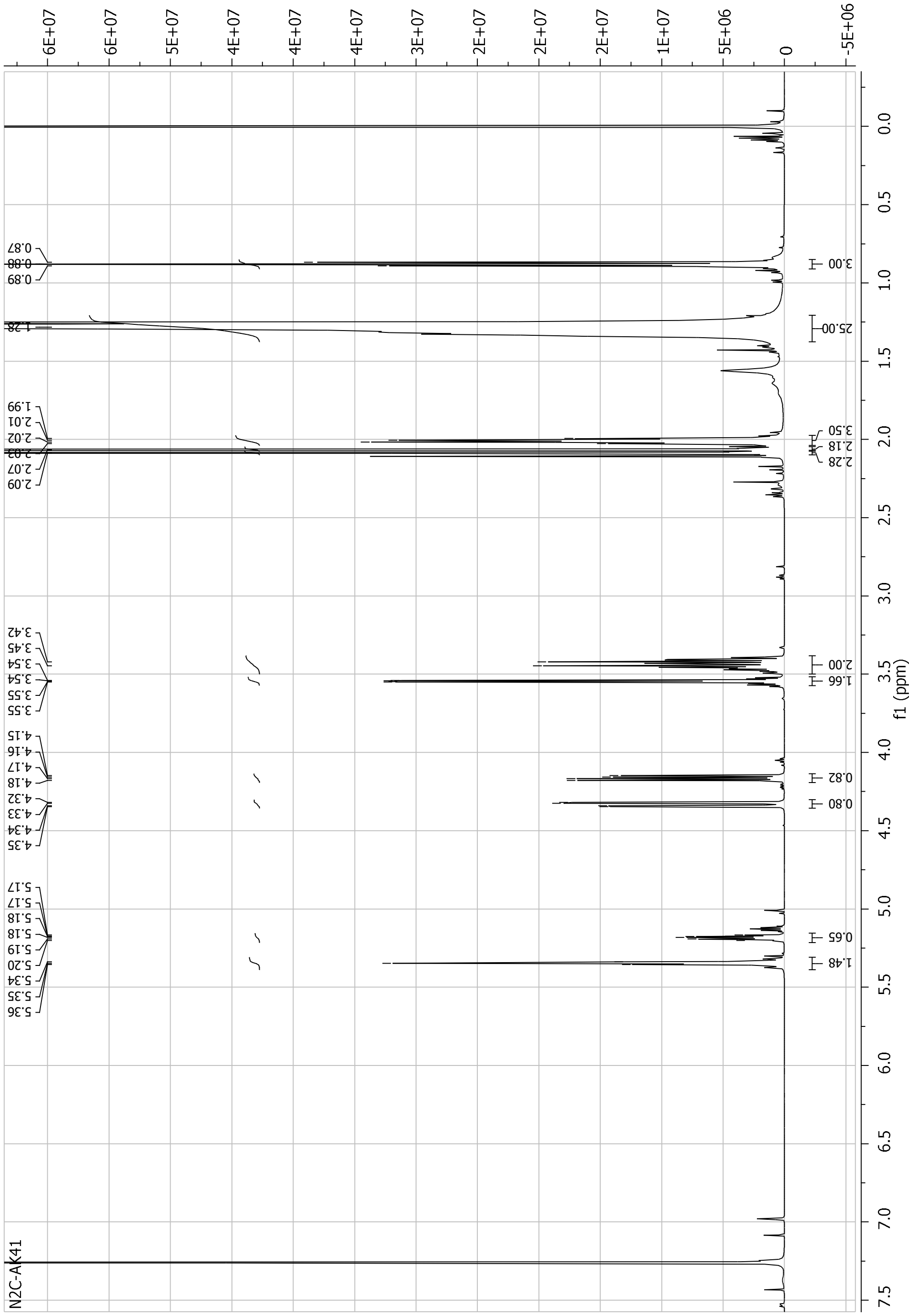

Figure SI1-5. ^{1}H NMR ($CDCl_3$) spectrum of compound **5f**.

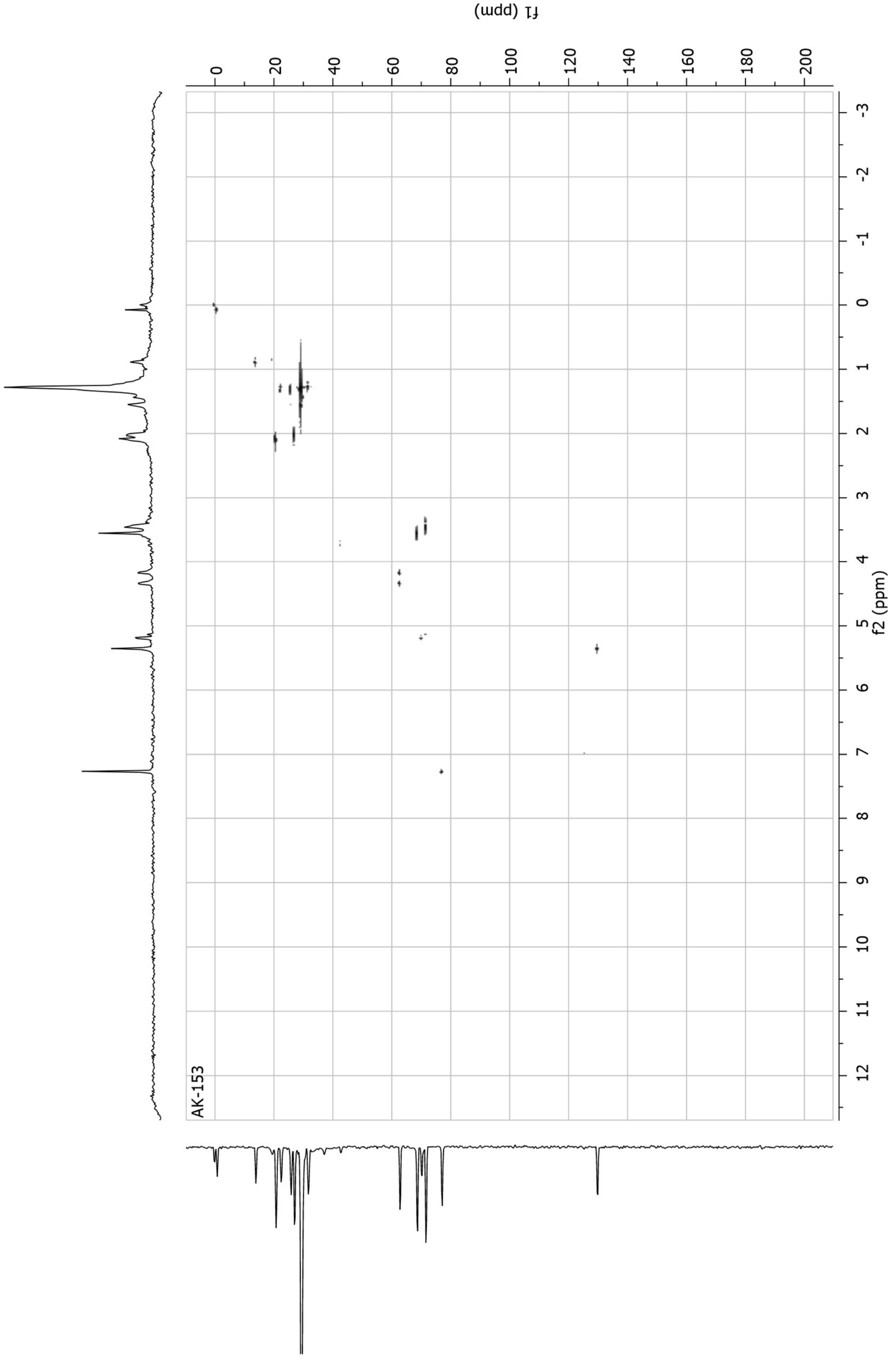

Figure SI1-6. 2D NMR HSQC ($CDCl_3$) spectrum of compound **5f**.

3-(eicosyloxy)propane-1,2-diacetate **5h**

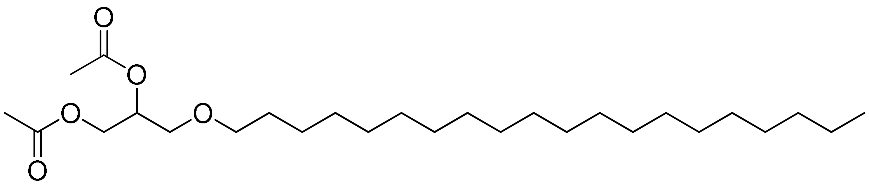

5h

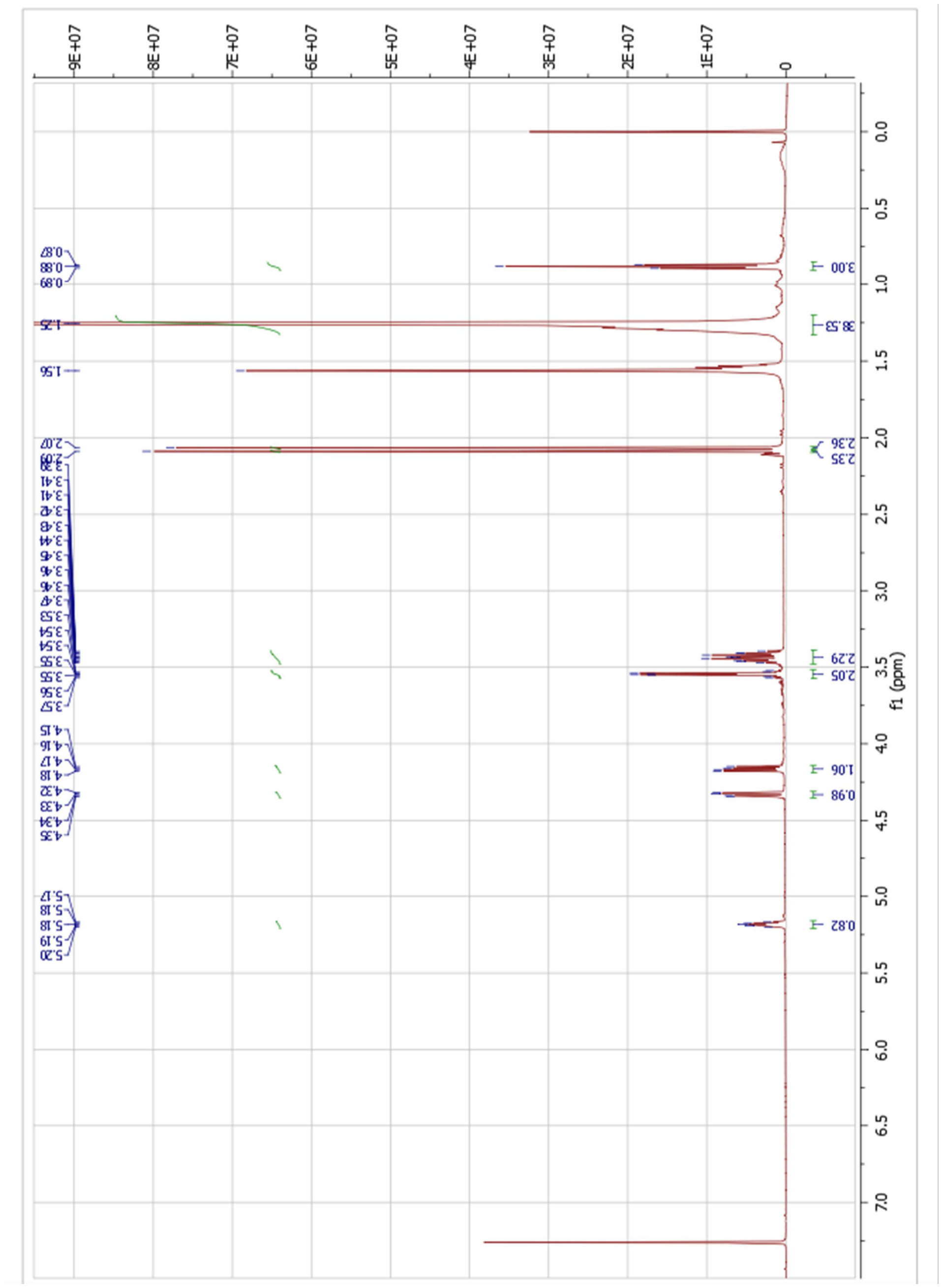

Figure SI1-7. ^1H NMR ($CDCl_3$) spectrum of compound **5h**.

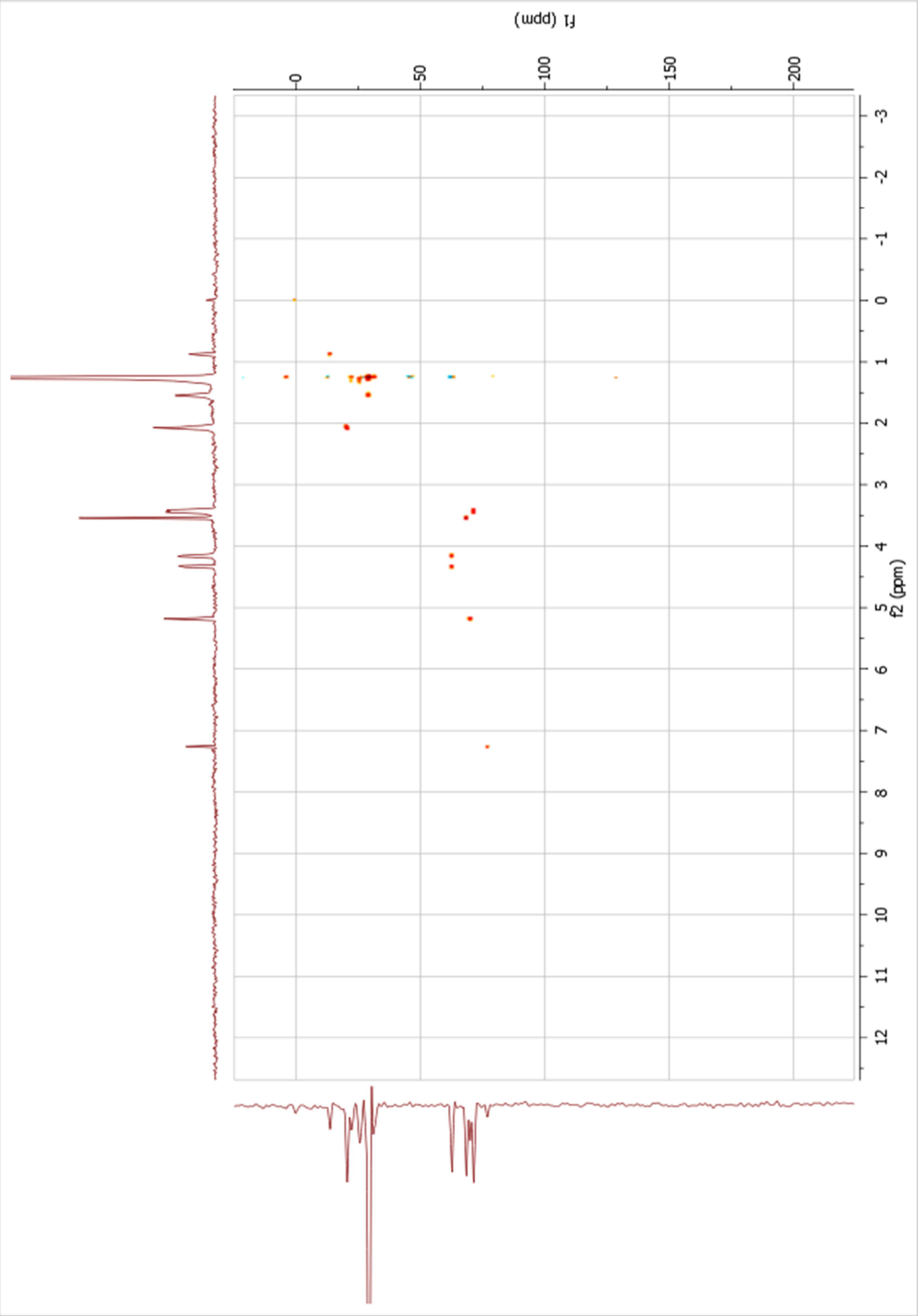

Figure SI1-8. 2D NMR HSQC ($CDCl_3$) spectrum of compound **5h**.

2. Chromatographic Characterization of Synthesized Compounds

GC-MS chromatogram of synthesized alkyl-glycerolipids

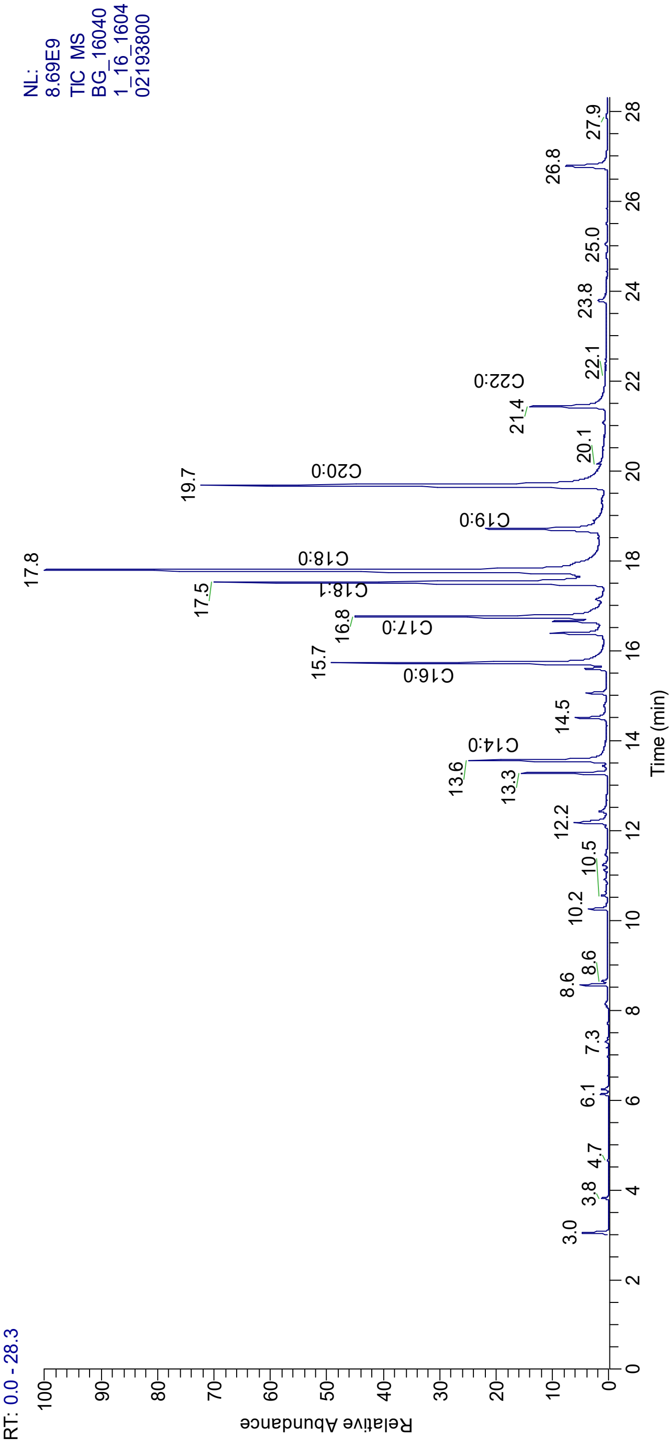

Figure SI2.1. GC-MS chromatogram of a mixture of synthesized alkyl-glycerolipids.

Alkyl chain R =	Retention time (min)	Molecular Weight ($g\cdot mol^{-1}$)	Most characteristic peaks (m/z, [%])
$C_{14}H_{29}$ (C14:0)	13.6	372.54	43 [base peak], 57 [60], 71[35], 83[30], 97[26], 111[13], 159[5]
$C_{16}H_{31}$ (C16:1)	15.0	398.58	43 [base peak], 57 [63], 69 [44], 95 [39], 117 [31], 159 [6], 222 [15]
$C_{16}H_{33}$ (C16:0)	15.7	400.59	43[base peak], 57[62], 71[38], 83[28], 97[27], 111[14], 159[7] 255[<5]
$C_{17}H_{35}$ (C17:0)	16.8	414.62	43[base peak], 57[62], 83[28], 97 [27], 111[15], 159[6]
$C_{18}H_{35}$ (C18:1)	17.5	426.63	43 [base peak], 55[52], 67[48], 81[52], 95 [44], 109[24], 117[18], 123[12], 135 [6]
$C_{18}H_{37}$ (C18:0)	17.8	428.65	43[base peak], 57[70], 71[45], 85[34], 97[33], 111[18],117[25], 125[8], 159[15], 283 [6], 325[6]
$C_{19}H_{39}$ (C19:0)	18.7	442.67	43[base peak], 57[65], 83[38], 71 [38] 97 [36], 111[20], 159[6]
$C_{20}H_{41}$ (C20:0)	19.7	456.70	43[base peak], 57[70], 71 [42], 83[36], 97 [36], 111[18], 117 [18],159[12], 125 [10]
$C_{22}H_{45}$ (C22:0)	21.4	484.75	43[base peak], 57[70], 71 [38], 83[28], 97 [38], 111[22], 117 [12], 125 [12] 159[6]

Figure SI2.2. Mass spectroscopy data.
